# A tri state mechanism for oxygen release in fish hemoglobin: Using *Barbus sharpeyi *as a model

**Published:** 2014-06

**Authors:** Mohammad Reza Dayer, Mohammad Saaid Dayer, Ali Akbar Moosavi- Movahedi

**Affiliations:** 1 Department of Biology, Faculty of Science, Shahid Chamran University, Ahvaz, Iran; 2 Department of Parasitology and Medical Entomology, Tarbiat Modares University, Tehran, Iran; 3 Institute of Biochemistry and Biophysics, University of Tehran, Tehran, Iran

**Keywords:** *Barbus sharpeyi*, Hemoglobin, Tri State Mechanism

## Abstract

Hemoglobin is a porphyrin containing protein with an α2β2 tetrameric structure and like other porphyrin compounds shows spectral behavior of species specific characteristics. Researchers tend to relate bands in the hemoglobin spectra to certain structural and/or functional features. Given the fact th↔at hemoglobin is the main oxygen carrier in animals functioning through the Oxy↔Deoxy equilibrium, the determination of oxy and deoxy conformations of hemoglobins of different animals may shed light on their oxygen binding properties. Absorption spectra at 280 and 373nm have been widely used to quantitate the formation of hemoglobin deoxy conformation. In the present work, however, we used an optical density ratio of OD373/OD280 as an index for deoxy formation. This ratio was determined for *Barbus sharpeyi *and human hemoglobins at different SDS concentrations, pH levels and temperatures to compare them from a structure-function point of view. Our data showed that under low concentrations of SDS (<2mM) *Barbus sharpeyi *hemoglobin folds in a tri-state pattern while human hemoglobin folds through a two-state phenomenon. This finding indicates that in contrast to those of other non aquatic animals, the hemoglobin of *Barbus sharpeyi *has a loosely folded tetrameric structure with remarkably more oxygen affinity

## INTRODUCTION

Hemoglobin is an oxygen binding protein predominantly found as α2β2 tetramer in different animals including humans and fish [1]. Hemoglobin has UV-Vis spectra similar to that of porphyrin, as it contains porphyrin prosthetic groups [2, 3]. These spectra comprise weak bands at the 450-650 nm region which are denoted as Q bands including a sharp and strong band in near-UV at about 412 nm called B or Soret band and N, L, and M bands at far-UV region from 200 to 400 nm [4, 5]. There are also other bands at far-UV regions which belong to the electronic transition of aromatic residues in hemoglobin sequences which interfere and mix with N, L and M bands [6, 7]. Researchers have provided evidence to justify assigning bands to far UV regions to certain transitions in heme groups or aromatic residues and relating them to structural features of the hemoglobin protein [8, 9]. In fact, micro-environment changes of aromatic residues or heme groups may result in spectral changes in far-UV regions. Therefore, the pattern of band changes may serve as an indication for specific alterations in tertiary and quaternary structures most often in R↔T transitions during hemoglobin oxygenation and deoxygenation [10, 11]. Among aromatic residues of hemoglobin, Trp (λmax=280 nm) shows higher absorptive coefficients than Tyr (λmax=274.6 nm) or Phe (λmax=257.4 nm) and hence contributes more to absorption spectra in the far UV region [10].

Trp37 of beta chains and Tyr42 of alpha chains are placed in α1β2 and α2β1 interfaces[12]. These interfaces (but not α1β1 and α2β2 interfaces) constitute the contact point of alpha and beta chains which undergoes alterations during oxygenation ordeoxygenation. Upon deoxygenation, non-covalent interactions of α1β2 and α2β1 interfaces increase and cause hemoglobin to accept a more folded conformation [7]. This over folding of hemoglobin causes Trp37 and Trp42 as well as two c-terminus residues, Tyr145 in beta and Tyr140 in alpha chains, to move to a more buried position which leads to a detectable decrease in absorption at 280 nm [8, 13].

Accordingly, the decrement of absorbance at 280nm may be used as an auxiliary index to ascertain the conversion of oxy hemoglobin to its deoxy state [14]. On the other hand, it has been demonstrated that proximal histidine moves away from heme iron upon deoxygenation [15]. This movement can result in a spectral change elucidated by an increase of absorbance at 373 nm. It is also reported that the protonation of the proximal histidine may lead to a complete dissociation of histidine from the ferrous ion of heme groups and hence produce a maximum absorbance increase at 373 nm [15-17]. In the present work, we showed that by combining the absorbance values of 280 and373 nm wavelengths in a ratio of OD373/OD280, a better index may be obtained to trace the changes of hemoglobin tertiary and quaternary structures even at small scales.

Circular dichroism spectra of deoxy hemoglobin show a sharp and negative band at287 nm. This band is a specific characteristic of the tens or deoxy conformation of hemoglobin [10, 11]. Despite global similarities between structures of the tetrameric hemoglobin of different animals in terms of the architecture of their subunits and mechanism of action, they have fine differences in terms of protein structures and mechanistic details of actions. These differences help animals to survive various environmental conditions and adapt to specific oxygen availability [18-20].

In order to adapt to low oxygen pressure in water, especially at high temperatures or acidic pH conditions, fish have evolved different mechanisms for oxygen uptake. These mechanisms include developing different hemoglobin isoforms with elevated oxygen affinity and the capacity to unload oxygen in acidic pH (Root effect instead of Bohr effect) in addition to their capability to rearrange in oligomerization states (from tetramer to dimmer or monomer) with higher oxygen affinity in threatening conditions [21, 22]. Change in ATP and GTP concentrations is an alternative mechanism regulating oxygen release from fish hemoglobin so that increased concentrations of ATP or GTP reduce hemoglobin affinity for oxygen and facilitate oxygen release [23,24].

In our previous work we have shown that in contrast to that of humans, *Barbusgrypus *hemoglobin has a loosely folded tetrameric structure [25]. We therefore postulated that at low oxygen pressure, this kind of structure may dissociate from tetrameric to dimeric and ultimately monomeric forms with more oxygen affinity [25]. We also showed that low concentrations of sodium dodecyl sulphate at pH 7 increased hemoglobin folding, converting the oxy hemoglobin to a deoxy form [26, 27]. In the present work, however, using *Barbus sharpeyi *as a fish model that processes hemoglobin of a more stable quaternary structure, we studied, in more detail, the changes that SDS imposed on hemoglobin structure, in hope of differentiating the responses of fish and human hemoglobins to structural alterations. We also examined the hypothesis regarding whether different responses of the hemoglobins to SDS treatment helped elucidate different architectures of *Barbus sharpeyi *and human hemoglobins.

## MATERIALS AND METHODS

Sodium n-dodecyl sulfate (especially pure grade) was obtained from Sigma. CM- Sephadex and Sephadex G-25 were from Pharmacia fine chemicals. Other reagents were of analytical grade. All solutions were prepared using double dist illed water.

**Sample Collection:** *Barbus sharpeyi *(BS) blood samples were obtained from caudal veins of healthy fish cultured in a pool at Azadegan culturing center in Ahvaz (Khuzestan Province, Iran). Fish were anesthetized by a sharp blow to the cranium and blood was collected using disposable syringes containing 0.2 ml saline buffer with 0.9% NaCl in 50 mM Tris-HCl pH 8 and 0.2% D-glucose and EDTA. Human adult hemoglobin was prepared from red blood cells of healthy donors [28-30].

**Hemoglobin Extraction** *: Barbus sharpeyi *and human erythrocytes were prepared at 0°C, washed three times with saline buffer containing 0.9% NaCl, 50 mM Tris-Hcl pH 8 and 1mM EDTA and centrifuged at 3000 rpm for 10 min. The washed, packed cells were lysed with an equal volume of 50 mM Tris-HCl pH 8.5. The stroma was removed after 1hour at 0°C by centrifugation at 10,000 rpm for 25 min [28]. Hemoglobin samples were striped of organic phosphate according to Riggs's procedure [29] by applying them to a Sephadex G-25 (1.5×60cm) column equilibrated with 100 mM Tris-HCl pH 8.2. Elution was carried out by a 0.1 M phosphate buffer pH 8 at a flow rate of 25 ml per hour [30]. Using a salt gradient eluent, CM-Sephadex cation exchanger at pH 6.5 and DEAE-Sepharose anion exchanger at pH 8.5 were utilized for further purification of human and BS hemoglobin respectively [28].

**Spectroscopic Experiments: **The concentrations of oxy- deoxy- and met- forms of hemoglobin were determined using a Shimadzu model UV-3100 (Japan) spectrophotometer and a thermostatically controlled cell compartment with Haak D8 water bath. To this end, the method described by Taylor [21] was used with minor modifications of equations at pH 7.0. An aquomet derivative of BS hemoglobin is prepared by reacting the oxy derivative with a three-fold molar excess of K3Fe(CN)6 for about 1/2 hr on ice. Removal of unreacted ferricyanide can be accomplished with gel filtration on sephadex G-25.

Circular Dichroism (CD) spectra of human and BS hemoglobins were recordedusing an Aviv model 215 Spectropolarimeter (Lakewood, NJ, USA). The concentration of the hemoglobin samples was 2mg/ml in a phosphate buffer (100 μM, pH 7). For CD spectroscopy, hemoglobin samples with 2 mg/ml concentrations were prepared by dissolving them in a phosphate buffer (pH 7 and 50mM concentration) [31]. The samples were then heated for denaturation at a constant rate of 80K/h and the CD signal was recorded at 222 nm as the θ222 function against temperature.

**Calculation of Equivalence Point: **Equivalence or stoichiometric point for hemoglobin deoxygenation upon titration by SDS, i.e. the SDS concentration required to exert a certain folding state, could be determined by plotting the first derivation of the curve OD373/OD280 against SDS concentrations. In this method, the equivalence point of each transition is the SDS concentration which causes a sharp inversion to the corresponding curve slope [32].

**Animal Welfare Committee: **The experimental procedures were conducted with due attention to bioethical guidelines approved by the Research Ethical Committee of Shahid Chamran University for the care and use of laboratory animals.

## RESULTS AND DISCUSSION

**Figure 1:**
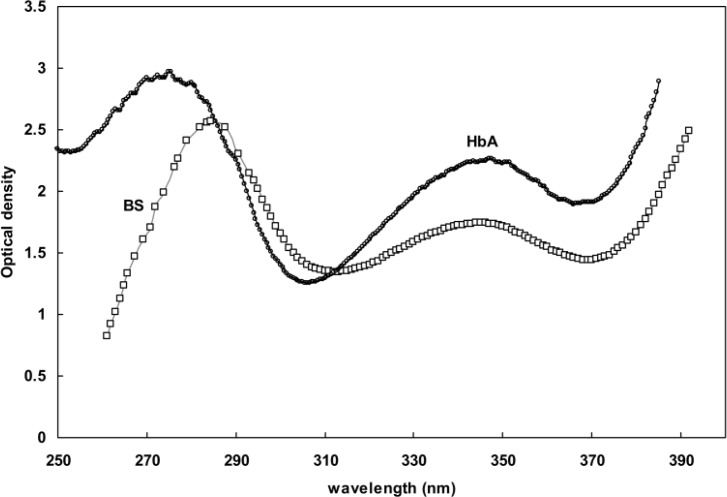
UV spectra of BS and human hemoglobin obtained in phosphate buffer (pH 7 and 50 mM at 37°C)

Figure 1 shows the UV spectra of human and BS hemoglobins in the range of 250-400 nm. There are two bands at about 270 and 350 nm. These bands are the combination of N, L and M bands of heme groups as well as the bands belonging to electronic transitions of aromatic amino acids. This region of the hemoglobin spectra consists of wavelengths considered to be characteristic of certain structural alterations in the hemoglobin protein. The increase in absorbance at 280 nm is proportionate to the extent of the aromatic groups' exposure to the surrounding environment and *vice versa*. Also, the decrease in absorbance at 373 nm is proportionate to the extent of proximal histidine displacement from its binding site to the heme group. Therefore, any absorbance increase at 280 nm or decrease at 373 nm could be interpreted as an indication of hemoglobin unfolding.

**Figure 2:**
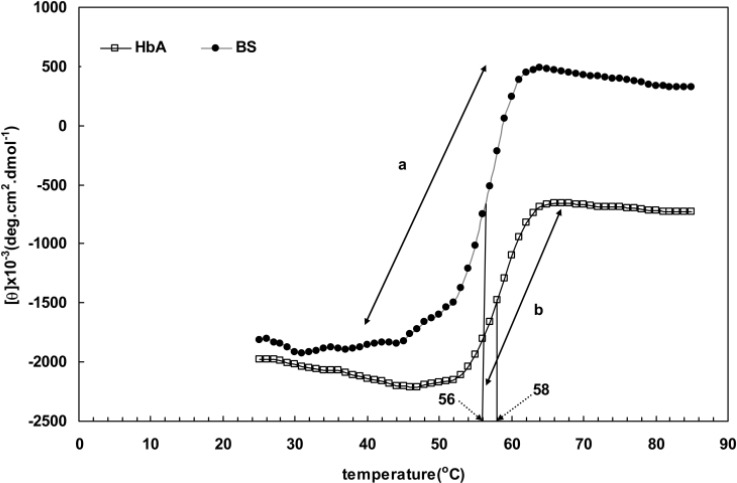
The far CD spectra of BS and human hemoglobin (0.2 mg/ml Hemoglobin dissolved in 50 mM phosphate buffer at pH 7.3 and 37°C

Figure 2 depicts the far UV circular dichroism spectra of BS and human hemoglobin samples in the range of 190 to 260 nm. These spectra are used routinely to determine protein secondary structures. Table 1 presents the details of hemoglobin secondary structures. As shown, the main secondary structure of hemoglobin is the alpha helix structure which comprises more than 49% of the regular secondary structures of hemoglobin. Table 1 also indicates a slight difference between BS and human hemoglobins in terms of helicity, with 2% more helicity for BS in the absence of SDS.

**Table 1:** Secondary structures of human and fish hemoglobin extracted from far-UV spectra obtained in the presence and absence of 1.5 mM SDS concentrations in 50 mM phosphate buffer pH 7.3 at 37°C

	**Alpha**	**Beta**	**Random ** **coil**
Fish	62.4	20.48	19.28
Fish+SDS	49.09	22.25	25.20
HbA	60.40	21.21	18.84
HbA+SDS	50.15	22.55	24.10

**Figure 3:**
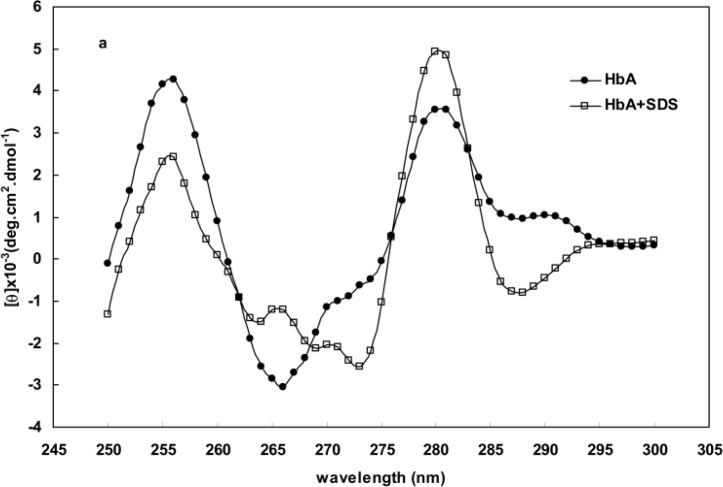
thermal denaturation of fish and human hemoglobin monitored by far-UV CD spectroscopy and presented as θ222(T) curve in 50 mM phosphate buffer, pH 7.3

Table 1 also indicates that the addition of a 1.5 mM concentration of SDS decreases the magnitude of alpha helix structures to about 13% and 10% in BS hemoglobin and human hemoglobin respectively. This finding conveys that fish hemoglobin is more sensitive to structural alteration by SDS. Thermal denaturation of human and BS hemoglobins is a useful method of studying their comparative structural changes. As shown in Figure 3, the fluctuation points of ellipticity curves against temperature could be considered as protein melting points. The Figure shows that the melting temperature of BS hemoglobin is about 2 degrees centigrade lower than that of human hemoglobin (56°C against 58°C). The next valuable evidence is the extent of increase in ellipticity of BS hemoglobin caused by thermal denaturation. As depicted, the increase in ellipticity for BS hemoglobin (arrow **a) **is about 1.5 times larger than that of human hemoglobin (arrow **b)**. This finding reveals the loosely folded structure of BS hemoglobin as well as its reduced resistance to thermal denaturation in contrast to human hemoglobin.

**Figure 4:**
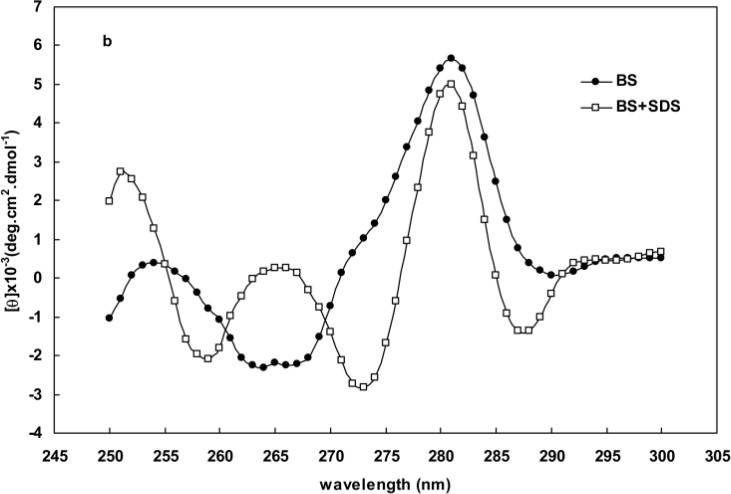
CD spectra of human hemoglobin (curve **a**) and BS hemoglobin (curve **b**) in the absence and presence of 1.5 mM SDS

Figures 4a and 4b represent the CD spectra of human and BS hemoglobins in the range of 250-300nm respectively. Some distinct changes are observed to be taking place in the CD spectra for oxy hemoglobins (in the absence of SDS) and deoxy hemoglobin (in the presence of 1.5 mM SDS) in the 280-290 nm region. Another spectral change seen at about 265 nm wavelength is the formation of a positive peak during the deoxygenation of hemoglobin which is characteristic of the deoxy species hemoglobin. This peak is not observed within the same range of spectra for other proteins.

**Figure 5:**
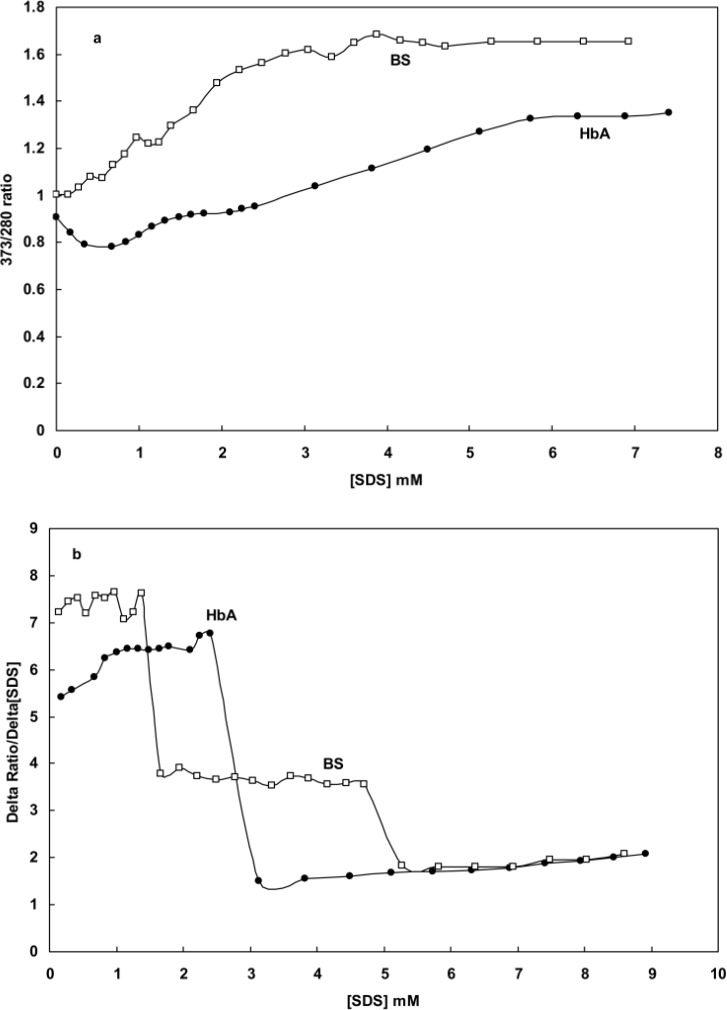
**a)** the fluctuation of $\frac{{\mathrm{OD}}_{373}}{{\mathrm{OD}}_{280}}$ ratios obtained for BS and human hemoglobin samples in phosphate buffer (50mM and pH7 at 37°C), **b)** The plot of calculated $\frac{∆\mathrm{ratio}}{∆[\mathrm{SDS}]}$ from the of graph a against SDS concentration.

Figure 5a shows the change in the ratio of OD373/OD280 upon SDS addition. Visual exploration of the plotted curve reveals the existence of multiple transition steps which may correspond to multi-phase changes occurring in the hemoglobins' structures. In order to gain clear insight into the probable transitions, we calculated the first derivative of the curve and plotted it as ∆ratio/∆[SDS] against SDS concentration in Figure 5b. As shown, there was a two-state transition (with two equivalence points) for human hemoglobin and a tri-state transition (with three equivalence points) for BS hemoglobin, suggesting the same structural transition in hemoglobin molecules.

**Figure 6:**
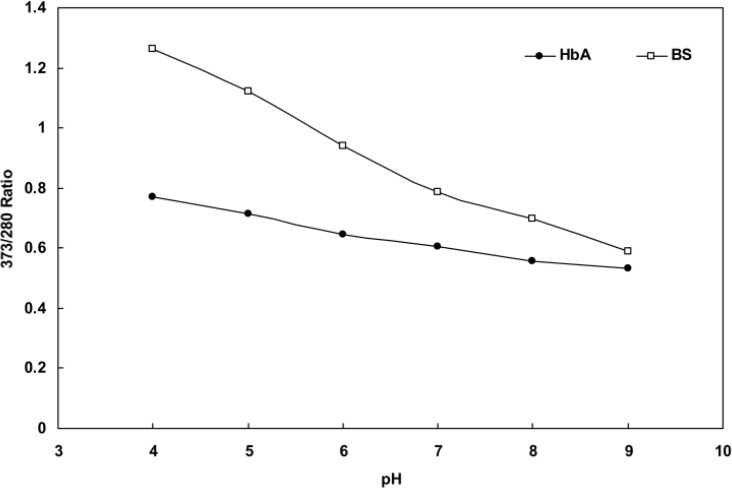
the plot of $\frac{{\mathrm{OD}}_{373}}{{\mathrm{OD}}_{280}}$ ratios obtained at different pH for BS and human hemoglobin

Figure 6 illustrates the changing pattern in the ratio of OD373/OD280 concomitant with increased pH for human and BS hemoglobins. As shown, the ratio of fish hemoglobin declines sharply as pH increases, dropping to a half of its initial value at pH 9, whereas that of human hemoglobin shows a steady decrease reaching a fourth of its initial value at the same pH. Once again, this finding confirms a more sensitive structure for fish hemoglobin compared with human hemoglobin in response to pH change.

The Far-UV spectra provide valuable information about aromatic residues contributing to a protein structure [18, 20-23]. Perutz et al. [33, 34] showed the formation of a negative peak at 287 nm upon the deoxygenation of hemoglobin. The data obtained from absorption essays, Far-UV CD and spectroscopy, such as those presented in Figures 1 and 4, indicated that the structural changes of hemoglobin during oxy/deoxy conversion can be visualized using 280 and 373 nm wavelengths [14-15]. However, we showed that the combination of both wavelengths in a ratio of OD373/OD280 provides better magnifying tool for small changes in the protein structure caused by deoxygenation of hemoglobin. Despite exhibiting higher helicity (Fig. 2 and Table 1), BS hemoglobin shows lower thermal stability than human hemoglobin, the melting temperature of BS hemoglobin being 2 degrees centigrade lower than that of human hemoglobin (Fig. 3). In our previous work, we reported a more loosely folded tertiary structure for fish hemoglobin, emphasizing its more extended topology for tetrameric structures compared with human hemoglobin [25]. To study the extent of susceptibility of the hemoglobin structures to a denaturant exposure, we determined the ratio of OD373/OD280 under increasing concentrations of SDS. As shown in Figure 5a, the general trend of non-linear increases in OD373/OD280 ratios relative to increases in SDS concentrations not only confirms the conversion of oxy to deoxy and ultimately to denatured conformations, but also verifies the occurrence of different transition states for hemoglobins as they interact with SDS. In order to quantitate these transitions, we obtained the first derivative of the OD373/OD280 curve and plotted it as a ∆ratio/∆[SDS] curve against SDS concentrations in Figure 5b. The figure clearly indicated a two-state transition for human hemoglobin and a tri-state transition for fish hemoglobin. Given that at neutral pH, SDS interacts with negatively charged hemoglobin exerting repulsive forces on its negative groups, the exposure to SDS causes hemoglobin to become more folded and to convert to a deoxy form [26, 27].

We, therefore, hypothesize that upon SDS addition, human hemoglobin in the first transition step becomes increasingly folded until the maximum folding is reached; aprocess during which the ratio of OD373/OD280 increases probably as a result of a reduction in absorbance at 280nm. In the second transition step and upon further SDS addition, the structural alteration of hemoglobin occurs at a lower scale since the increase in OD373/OD280 ratio is driven mainly by the displacement of proximal histidine. In fact, histidine displacement resulting in the breakage of the proximal histidine bond to heme groups results in increased absorbance at 373 nm. Nonetheless, the magnitude of such increase and its effect on OD373/OD280 ratio remains lower than what actually occurred due to hemoglobin folding in the first transition step.

In the case of fish hemoglobin, however, there are two separate phases with three transition steps. In fact, the addition of SDS enhanced the folding of the fishhemoglobin structure in two separate phases. In the first phase, SDS caused the loosely folded structure of fish hemoglobin to fold into a structure similar to that of human hemoglobin as illustrated in Figure 5b. This first transition step involved the formation of more compact tetramers at subunit levels with reduced absorbance at 280nm. Further addition of SDS to such structures triggered the second phase with two distinct transition steps similar to those of human hemoglobin. To provide confirmatory data for such a mechanism, we tested the ratio of OD373/OD280 for fish and human hemoglobins at different pH levels. The recorded data, as depicted in Fig. 6 reveals two important points; the first suggesting that acidifying the protein (low pH) causes a sharp increase in the OD373/OD280 ratio and the second showing that the OD373/OD280 ratio line of fish hemoglobin has a steeper slope than that of human hemoglobin. This finding provides further evidence supporting the higher susceptibility of fish hemoglobin to pH alteration compared with human hemoglobin, which can probably be attributed to its loosely folded structure. Given the following equilibrium:

*HHb+ *4O2 *↔Hb *( *O *)4+ 4H^+^

it is known that decreasing the pH from its neutral level shifts the equilibrium to the left, dissociates oxygen from hemoglobin and converts it to deoxy hemoglobin with a more compact structure [19, 20]. Under such circumstances, a decrease of absorbance at 280 nm and therefore an increase in the OD373/OD280 ratio is expected as discussed above. Furthermore, it has been reported that in acidic conditions, proximal histidine becomes protonated and dissociated from heme groups, leaving them as four coordinate groups with a distinct band at 373 nm [15].

This report provides future fortification to our argument regarding the less folded structure of fish compared to human hemoglobin. Our present and previous findings suggest that fish hemoglobin possesses a loosely folded structure of higher performance and greater oxygen affinity, enabling fish to adapt to various threatening environmental pollutants such as denaturants, acidic conditions or increasing temperatures. The structure of fish hemoglobin shows a relatively faster response and reactivity to harsh conditions by predominantly dissociating to a monomeric form of higher oxygen affinity to meet the animals' demand for oxygen.
